# High Flow Nasal Cannula, Is There a Role in COPD?

**Published:** 2017

**Authors:** Nicholas S Hill

**Affiliations:** Tufts Medical Center, Boston, MA USA

High Flow Nasal Cannula refers to the delivery of gas intra-nasally via loose-fitting cannulae at flows up to 60 l/min and with FIO2 that can be varied between room air and 100%. The modality is generally well tolerated because the nasal cannulae are comfortable and the gas is heated and humidified to body temperature and saturation. In addition, it doesn’t interfere with speech or eating. Physiologically, it has a number of advantages over standard oxygen, including the humidification that enhances ciliary action and secretion removal, high inspiratory flow that cuts down on entrainment of room air, thus ensuring a more reliable delivery of a targeted FIO2, flushing out of upper airway dead space that improves efficiency of ventilation, reduction of respiratory rate that helps to cut down on work of breathing per minute, and a small amount of positive end expiratory pressure that may help to counterbalance auto-PEEP. In addition, it is clearly better tolerated than either standard mask oxygen delivery systems or noninvasive ventilation.

The modality has seen increasing use in acute care settings throughout North America and Europe because of emerging evidence that it works at least as well as noninvasive ventilation in postoperative and postextubation settings. It may even outperform NIV in patients with pneumonia and ARDS by lowering intubation rate in more hypoxemic patients (P/F < 200) and also ICU and 90d mortality. Although the role of NIV in treating COPD exacerbations is well established in treating COPD patients with acute hypercapnic respiratory failure (AHRF) has been well established, it still fails in 10–25% of patients, partly because of intolerance to the mask. Whether HFNC may have a role in supplementing the use of NIV in treating AHRF in COPD has not been determined. Physiologically, it may be quite helpful by decreasing WOB, facilitating secretion removal, and providing PEEP. It also is likely to be better tolerated than NIV.

We have initiated a study to evaluate the effects of varying settings of HFNC on respiratory rate, tidal volume, diaphragmatic work of breathing and gas exchange in patients with severe COPD as well as describe the subjects’ comfort and dyspnea. We enrolled subjects with chronic respiratory failure from severe COPD (GOLD stage III or IV) and placed them on various levels of HFNC and NIV in random fashion while recording respiratory Inductive plethysmography to measure tidal volume, respiratory rate, and inspiratory time on a breath by breath basis. Esophageal and gastric manometry was used to measure transdiaphragmatic pressure and calculate the pressure time product of the diaphragm. Percutaneous CO2, heart rate, and pulse oxygen saturation and self-reported comfort with breathing were recorded. We used CPAP and BiPAP 10/5 as comparators. Results are presented in the Table below. Subjects found mask and airflow pressure of HFNC to be more comfortable than NIV and preferred HFNC. .

**Table 1.** The effects of varying settings of HFNC on respiratory rate, tidal volume, diaphragmatic work of breathing and gas exchange (^*^)

	Baseline	HFNC10L/min	HFNC30L/min	HFNC45L/min	HFNC60L/min	CPAP0	CPAP5	BiPAP10/5
Respiratory Rate Counted (breath/min)	13.87	12.28	10.45	11.02	9.50	14.42	14.07	12.79
Tidal Volume (mL)	422.14	552.89	508.41	518.86	519.57	603.76	528.23	572.41
Minute Volume (L/min)	5.86	6.79	5.31	5.72	4.93	8.71	7.43	7.32
Transdiaphragmatic Pressure (cmH20)	18.49	18.39	19.73	23.37	19.65	19.52	14.67	16.17
Pressure-Time-Product per minute (cmH20*sec)	134.16	129.05	108.96	120.12	89.34	151.34	105.94	102.40
Percutaneous CO_2_	44.71	43.96	43.73	44.41	43.79	45.36	44.04	47.39
Inspiratory Time/ Total breathing time	0.25	0.24	0.18	0.19	0.16	0.28	0.25	0.22

(^*^) Pisani L, Fasano L, Corcione N, Comellini V, Musti MA, Brandao M, et al. Change in pulmonary mechanics and the effect on breathing pattern of high flow oxygen therapy in stable hypercapnic COPD. Thorax. 2017;72(4):373–375.

We conclude that in patients with severe stable COPD (GOLD Stage III–IV), HFNC at flow rates ≥ 30L/min decreased respiratory rate and Ti/Ttot as well as diaphragmatic work of breathing per minute. NIV appears to lower minute volume in comparison to NIV and is deemed as more comfortable and preferable by patients. These results lay the foundation for trials looking at the role of HFNC to treat COPD exacerbations in an acute care setting but it is important to be cautious using this modality in patients with severe exacerbations until studies have examined it’s efficacy compared to NIV.

